# Modeling the Geometry and Filter Composite of the Air Cleaner

**DOI:** 10.3390/ma17204969

**Published:** 2024-10-11

**Authors:** Pola Kalina Olszewska, Justyna Pinkos, Dominik Borkowski, Maciej Jablonski

**Affiliations:** 1 Institute of Architecture of Textiles, Lodz University of Technology, 90-924 Lodz, Poland; 2 Lukasiewicz Research Network—Lodz Institute of Technology, 19/27 M. Sklodowskiej-Curie, 90-570 Lodz, Poland; dominik.borkowski@lit.lukasiewicz.gov.pl

**Keywords:** air purifier, computer simulations, filter component, hydrosol

## Abstract

Air pollution is currently the most significant environmental factor posing a threat to the health and lives of European residents. It is a key cause of poor health, particularly respiratory and cardiovascular diseases. The primary aim of the study was to numerically determine the impact of the air purifier model’s geometry on the distribution of air within a room and to conduct experimental tests on the filtration efficiency and preliminary antibacterial activity of filtration composites. The scope of the work included designing an air purifier model in the form of a pendant lamp and performing computer simulations in Ansys software to identify the optimal shape. The experimental research focused on developing filtration composites consisting of nonwoven fabric with an active hydrosol layer, meltblown nonwovens and a carbon filter. The study results showed that the SMMS composite with 50% thyme and carbon nonwoven exhibited the highest filtration efficiency for both small and large particles.

## 1. Introduction

Air pollution is currently the most significant environmental factor affecting the health and life risks of Europe’s population. It is a key cause leading to ill health, particularly respiratory and cardiovascular diseases. The key particles that threaten human health are fine particulate matter, ozone, nitrogen dioxide and carbon monoxide [1]. It might seem that locking ourselves indoors would avoid harmful substances in the air. However, it should be noted that there are numerous sources of pollution indoors, and air exchange is limited. People who stay indoors with polluted air are at risk of diseases such as stroke, lung cancer, ischemic diseases and chronic obstructive pulmonary disease [1,2,3]. Particulate matter (PM) is the most important determinant of indoor air quality as well as an indication of the level of air pollution. The U.S. Environmental Protection Agency has divided PM into three groups based on their size: PM10, or coarse particles PM < 10 μm in diameter, PM2.5, or fine particles PM < 2.5 μm in diameter, and PM1.0, or ultrafine particles PM < 0.1 μm in diameter. The smaller the particle, the greater its power to penetrate the human bloodstream and lungs. Not only does particle size matter for human health but also its chemical composition [2,4]. 

Another very dangerous substance for humans in polluted air is volatile organic compounds (VOCs), which account for as much as 60% of all atmospheric pollutants. This is a wide range of organic compounds with different physical and chemical properties, consisting of one or more elements of carbon and one or more elements of bromine, oxygen, nitrogen, sulfur, hydrogen, silicon and phosphorus. These compounds are characterized by their ability to rapidly change their state of aggregation into gas and vapor, their poor solubility in water and their rapid boiling at normal pressures of 50 °C to 250 °C, causing the compounds to already enter the air we breathe at room temperature. Due to the variety of VOCs, they affect human health in many different ways. Short-term exposure to VOCs can result in irritation of mucous membranes, headaches and dizziness, feelings of fatigue, nausea, vomiting and aggravation of symptoms of existing lung and nervous system diseases. Being exposed to volatile organic compounds for a prolonged period of time can result in damage to the nervous system, loss of consciousness, kidney or liver damage, the development of cancer and even death. When assessing their harmfulness, which depends on the intensity, TVOCs (Total Volatile Organic Compounds) are taken into account [5,6,7].

Filters in air purifiers are used to get rid of pollutants in the room to improve air quality. The devices remove particles from the air, i.e., dust, dander, mold spores, pollen and PM2.5 and PM10 suspended particles. They are used both in industry and at home. The structure of the purifier consists of a fan, a pre-filter in the form of a sponge or dense mesh, a carbon filter, a HEPA (High-Efficiency Particulate Air) filter. The pre-filter is responsible for capturing large pollutants, i.e., dander and dust, which could clog the next filters. Some purifiers additionally include an ionizer, UV lamp or humidifier [8]. Studies on reducing microorganisms from the air with indoor air purifiers have shown that the actual effectiveness of air purifiers is much lower than the manufacturer’s claimed values [9]. Studies of air purifiers for removal of micron-sized particles have also shown that most of the experimentally obtained CADR (Clean Air Delivery Rate) ratios were lower than the manufacturer’s claims [10,11]. 

Currently, computer simulation programs are used very often, studying selected physical, mechanical or thermal phenomena. Using computational fluid dynamics (CFD), the authors modeled the movement of airborne droplets released by individuals, focusing on a two-bed dormitory setup [12]. They analyzed different air purifier flow rates and positions to determine the most effective configurations for removing droplets from the air. The findings indicate that a larger airflow rate significantly enhances the droplet removal efficiency, and the position of the air purifier within the room plays a critical role in optimizing droplet reduction. Research on hanging devices, particularly in household conditions, is presented in article [13,14]. The CFD model is used to evaluate the effectiveness of air purifiers in various spatial configurations and different ventilation conditions. The models showed that the best results were achieved when the purifier was placed in the central part of the room, as it distributed the purified air evenly. It was concluded that the effectiveness of air purifiers is closely dependent on the room size and the placement of the device. Similar research presenting a CFD model for analyzing airflow and optimizing the placement of air purifiers to achieve the highest cleaning efficiency in various spatial configurations and ventilation conditions is presented in article [15,16]. The models considered different ventilation parameters, such as various positions of air inlets and outlets, as well as different ventilation speeds. The simulations showed that a key factor affecting the effectiveness of air purifiers is both their placement and interaction with the ventilation systems. Optimal placement of air purifiers, especially near central airflow points, significantly improved the efficiency of pollutant removal. The best results were obtained when the purifiers were positioned in locations where they could effectively cooperate with the natural airflow within the room. Interesting research presenting the modeling of an air quality monitoring network using sensors that allow the collection of detailed spatio-temporal data is discussed in article [17]. The network consisted of three sensors located in a seaside town in southern Italy, in points sensitive to human activity. The study found that low-cost sensors can significantly improve air quality monitoring by providing detailed spatio-temporal data. It was also argued that combining sensors in a network increases data utility, which is crucial for precise pollution mapping. Interaction with weather conditions is important in modeling pollution movement and assessing the impact on air quality.

The main objective of the work undertaken was to numerically determine the effect of the geometry of the purifier model on the way air propagates into the room, and to perform experimental tests of filterability and preliminary tests of the antibacterial activity of filter composites. The scope of work carried out included designing a model of an air purifier in the form of a pendant lamp and conducting computer simulations to select the optimal shape. Experimental research involved the development of filter composites, consisting of a nonwoven fabric with a hydrolat active layer, meltblown nonwovens and a carbon filter. It was assumed that such a composite should have appropriate antibacterial and filtration properties. At the first stage, the selection of nonwoven fabric for making the composite was made—spunbond type, meltblown type with suitable filtration properties, and the selection of nonwoven carbon filter. Next, spunbonded nonwoven fabrics were produced from PBS polymer, which were characterized in terms of physical–mechanical and performance properties. In the final step, suitable hydrolats with biocidal properties were selected, and two solutions of 50% and 100% concentrations were prepared. Using the surfacing method, layers of the selected solutions were applied to the spunbond nonwoven fabric. The filtration efficiency was then tested for each layer and the filter composites. 

## 2. Numerical Studies of the Geometry of Lampshades

**Figure 1:**
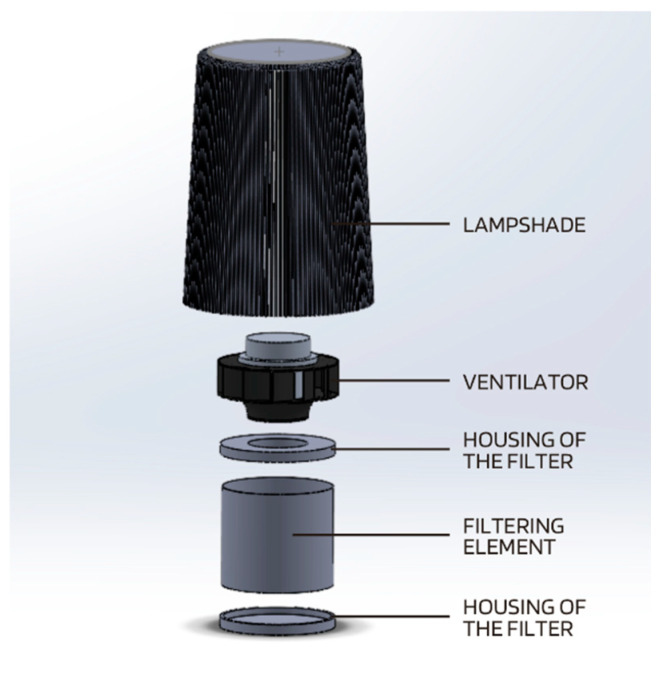
Elements of the geometric model of the air purifier.

Numerical studies were implemented in ANSYS CFX software. Before simulating the airflows through the numerical model of the lamp shade with the air cleaner, it was necessary to model it in SolidWorks software. In the first step of geometric modeling, the exact dimension of the filter element was defined, which, for the purposes of the simulation, combined all the filters: pre-filter, HEPA filter and carbon filter. The next step was to make a fan, adapted to the size of the filter made. The last part modeled was a housing in the form of a lamp shade. Next, a numerical model of the air purifier mounted in the ceiling lamp was made from all the abovementioned elements. Figure 1 shows all the geometric elements included in the air purifier and their location.

**Figure 2:**
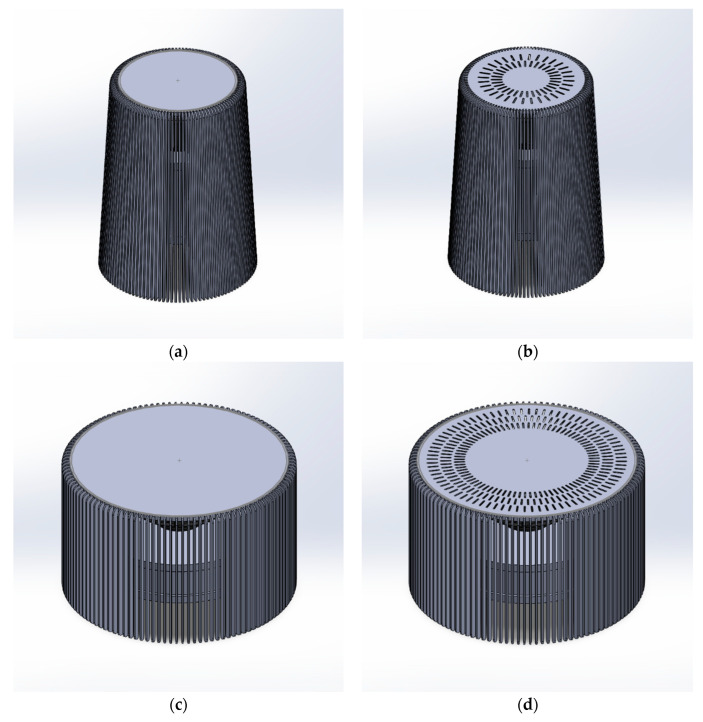
Variants of geometric models: (**a**) first, (**b**) second, (**c**) third, (**d**) fourth.

Four variants of numerical models were developed. The first and second variants had external dimensions: height H1,2 = 365 mm, diameter D1,2 = 275 mm. The diffuser consists of 120 rungs with dimensions: width Ds1,2 = 15 mm, thickness Ts1,2 = 3 mm, height Hs1,2 = 365 mm, the rungs were attached to the top of the lamp having dimensions: X1,2 = 206 mm, Y1,2 = 15 mm, at equal intervals (Figure 2a). The second variant had 64 additional air outlets located at the top of the lampshade in two rows, the inner radius from the center of the lampshade to the beginning of the first row was equal to R1 = 42 mm, the radius from the center of the lampshade to the beginning of the second row was equal to R2 = 65 mm, each outlet had dimensions: width D = 3 mm, length L = 20 mm (Figure 2b). The dimensions of the individual elements of the third (Figure 2c) and fourth variants were H3,4 = 270 mm, D3,4 = 436 mm. The lampshades consisted of 120 rungs with dimensions: Ds3,4 = 15 mm, Ts3,4 = 3 mm, Hs3,4 = 270 mm, attached to the top of the lamp X3,4 = 406 mm, Y3,4 = 15 mm, at equal intervals. The fourth lampshade contained an additional 240 holes for air outlet of the same dimensions as the second lampshade. The holes were located at the top of the diffuser in three rows, with the first inner radius being R1 = 113 mm, the second radius from the center of the diffuser R2 = 139 mm, and the third outer radius R3 = 165 mm (Figure 2d). 

The geometric models made were imported into the simulation software, then each was placed in a cube with all sides equal to 1 m, which acted as an air room. In the next step, the finite element mesh was adjusted by adopting a Hexa/Solid mesh with a finite element dimension equal to 12 mm. The air inlet was placed on the inner wall of the filter element, and its velocity was set at 5 m/s. The air outlet was assigned to the top wall of the cube, and a pressure equal to 0 Pa was set on it. The frequency of the fan was set at 1530 rpm in accordance with the current standards of air purifiers. In the final step, material parameters were defined for the filter elements in the model. A Young’s modulus of 3 GPa, a Poisson’s ratio of 0.3, and a porosity of about 90% were assumed [18,19]. For the housing, the parameters adopted were aluminum Young’s modulus of 70 GPa, Poisson’s ratio of 0.33, density of 2.700 kg/m^3^; and for the diffuser, the strength parameters for polypropylene (PP) Young’s modulus 1.5 GPa, Poisson’s ratio 0.42, density 910 kg/m^3^, tensile strength: 30 MPa [20,21].

## 3. Results from Numerical Studies

**Figure 3:**
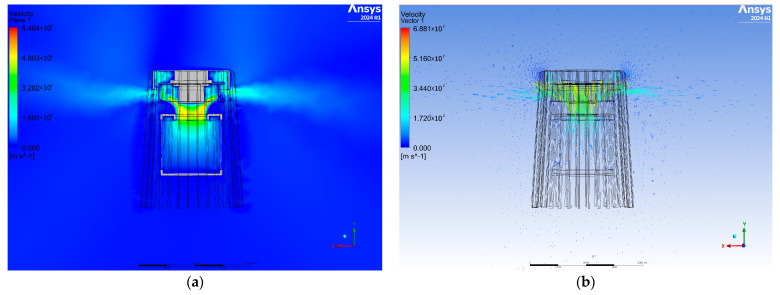
View of the cross-sectional plane for air velocity (**a**) and velocity vectors (**b**) for variant one.

The results of the numerical tests were analyzed in terms of air velocity and propagation pattern depending on the model geometry used. Figure 3 shows a cross-sectional plane view of the airflow velocity contours and velocity vectors for the first variant. A large amount of air accumulates in the upper part of the diffuser, and its flow is symmetrical. The air velocity increases at the entrance to the fan, which is the result of the fan blades, and decreases toward the outlet. The velocity vectors show the direction of airflow, which is largely perpendicular to the purifier, while some of the air is directed upward. 

**Figure 4:**
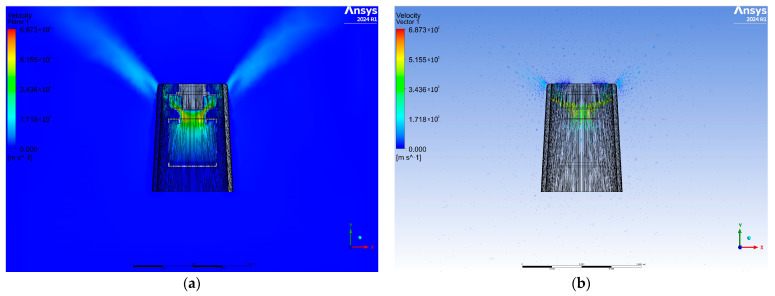
View of the cross-sectional plane for air velocity (**a**) and velocity vectors (**b**) for variant two.

In the next step, the results were analyzed for the second variant of the diffuser having openings from the top (Figure 4). It is noted that the airflows are also symmetrical to each other, and the highest air velocity is achieved at the entrance to the fan. The velocity vectors are marked on the same cross-sectional plane as the velocity contours and determine the direction and magnitude of the airflow. Higher values of air velocity were noted at the top of the purifier, near the air outlets, which suggests faster flow than at the bottom of the diffuser.

**Figure 5:**
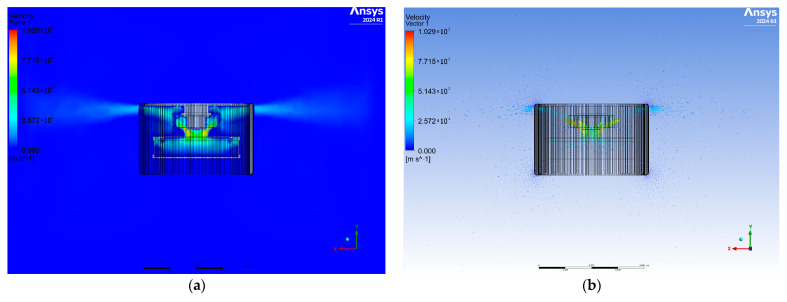
View of the cross-sectional plane for air velocity (**a**) and velocity vectors (**b**) for variant three.

Figure 5 shows the airflow simulation for the third variant. Also in this variant, it is noted that the airflows are symmetrical; however, the direction of the airflow is perpendicular to the purifier. The air reaches a higher velocity at the entrance to the fan and decreases towards the air outlets. The cross-section of the plane and the air velocity vectors placed on it show that in the upper part of the diffuser the vectors are longer than in the lower part.

**Figure 6:**
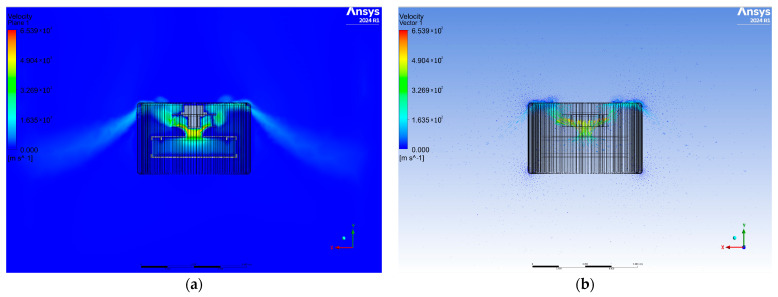
View of the cross-sectional plane for air velocity (**a**) and velocity vectors (**b**) for the fourth variant.

Figure 6 shows a view of the velocity contours of the airflow cross-sectional plane for the fourth variant. On both sides of the purifier, the air is arranged in a similar way. Some of the air accumulates in the upper part of the diffuser. The air velocity is highest at the inlet—to the fan and decreases as it approaches the contours of the diffuser. Air velocity vectors located on the mantle of the cross-section show that the velocity and direction of the flow accumulate in the upper part of the diffuser and then descend at a lower speed. 

The numerical studies conducted provided valuable information on the operation of the air purifier, showing how air is drawn in then mixed by the fan and blown out. For each simulation performed, it was noted that the air velocity increases at the entrance of the fan and decreases with the direction of exit. Each model tested distributed clean air symmetrically. Also, in all simulations it can be observed how important the openings in the top of the diffuser are for good air distribution. In models without additional outlets, the air is trapped in the center of the unit instead of flying out into the room. 

**Figure 7:**
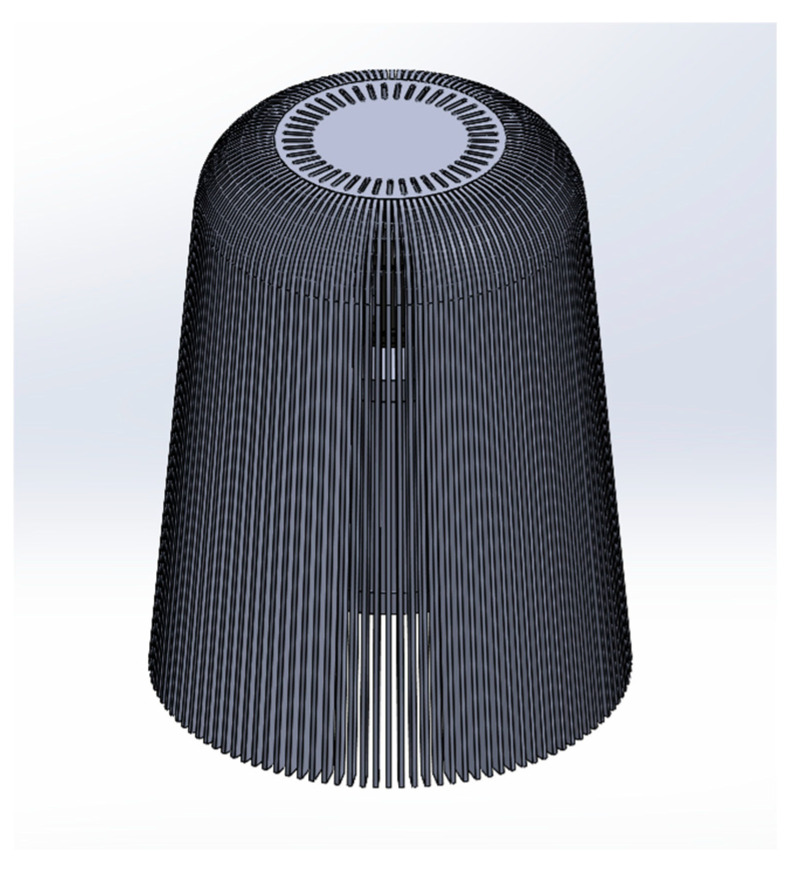
Final geometric model of the lampshade.

Variants one and two had wide air streams, which resulted in better distribution of clean air into the room. For variants three and four, the streams are narrower. This is a result of the wider diffusers, which causes the air to collect in their upper sections. For variant four, the upper outlets are an additional air outlet but also cause the direction of the air to be directed downward. Such flows could significantly reduce the comfort of the purifier, causing a draughty effect in the room. The fan speed set in the numerical tests is the fastest assumed speed, suggesting that variant four could insufficiently “far” distribute air at lower fan power settings. After analyzing all simulation results and considering the available options, it was decided to choose variant two of the diffuser. Its optimal shape allows for adequate air distribution to the room. Therefore, based on the computer simulations carried out, the final geometric model of the diffuser was developed (Figure 7). The geometrical model of the final lampshade had external dimensions: H = 367 mm, top diameter D1 = 235 mm, bottom diameter D2 = 290 mm and consisted of 120 rungs with dimensions: W = 15 mm, T = 3 mm, H = 367 mm, were attached to the top of the lamp at intervals, with dimensions: Xs = 140 mm, Ys = 15 mm. The model contained 45 additional air outlets located at the top of the lampshade, in one row, from the center of the lampshade to the beginning of the row the radius was r1 = 47 mm, the outlets had the same dimensions as for the other models with holes.

**Figure 8:**
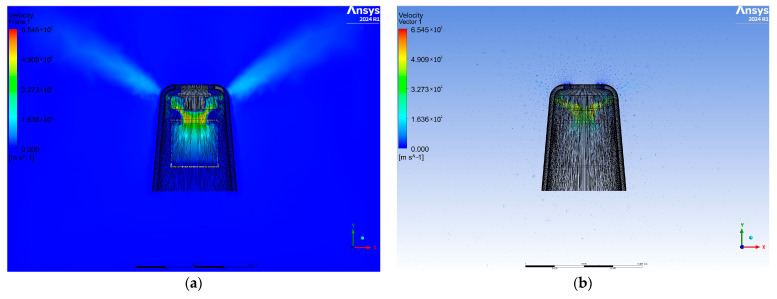
View of the cross-sectional plane for air velocity (**a**) and velocity vectors (**b**) for the final variant.

The same type of finite element mesh was generated for the developed geometric model, and exactly the same initial and boundary conditions for the simulation were adopted. Figure 8 shows a cross-section of the airflow velocity contour plane. The streams, located at the top of the model, are wide and spread out, ensuring adequate air distribution. The air velocity vectors are placed on the same cross-sectional plane. The vectors point upward at a higher velocity and descend slightly at a reduced velocity. 

## 4. Experimental Study of Filter Composite with Biocidal Properties

In an era of growing awareness of air pollution and the dangers of it, innovative solutions in the form of new filtration materials are needed. One of them could be the making of a filter composite that not only effectively removes pollutants from the air but also demonstrates the ability to neutralize trapped pathogens. Such a solution would make it possible not to use additional devices in air purifiers such as UV lamps, which are designed to eliminate pathogens, namely: bacteria, viruses and mold. Polybutylene Succinate (PBS) is a polymer obtained by polycondensation of succinic acid with butandiol. It is an aliphatic polyester that has properties similar to polypropylene. One of the main advantages of PBS is its biodegradability—the material breaks down into water and carbon dioxide. Another important advantage is its good mechanical properties and thermoplastic properties due to its linear structure, which makes it easy to process [22]. Hydrolats are obtained by distilling whole plants or only parts of them with steam. This phenomenon results in a product consisting of oil and water with dissolved volatile compounds. Hydrolats are much milder than essential oils, whose fragrance and effect are intensified. In a hydrolate, the concentration of the active ingredient varies from 0.01% to 0.04%. Plants such as thyme and witch hazel show strong biocidal properties against bacteria such as *Staphylococcus aureus* (*S. aureus*) and *Escherichia coli* (*E. coli*) in vitro [23,24]. 

In this article, the nonwoven meltblown filter fabric was not modified, as it could lose its filtration properties (load lifting) and structure The spunbond nonwoven fabric was used as a layer with a biocidal composite, which further enhanced the mechanical properties of the meltblown nonwoven fabric, making the filter more durable. The experimental study carried out was a preliminary analysis in terms of modern and environmentally friendly filter materials, which can find application in many industries.

### 4.1. Material

Nonwoven meltblown fabric made of polypropylene—from FIBECO s.c. (Poland). The basic parameters of the nonwoven fabric are shown in Table 1.

Nonwoven carbon fiber made of polyester from Aksfilter (Poland) in black, meets the PN-EN 779:2012E standard [25]. The basic parameters are shown in Table 2.

- nonwoven fabric made using the spunbond method with PBS;
- poly(succinate) butylene—PBS FZ71PM—from PTT MCC Biochem Co., Ltd. (Bangkok, Thailand),
- thyme hydrolate, manufacturer Institute of Natural Aromatherapy (Malaga, Spain);
- echinacea hydrolate from Ecospa (Warsaw, Poland);
- test strains from the American Pure Culture Collection (ATCC):
- *Staphylococcus aureus* ATCC 6538,
- *Escherichia coli* ATCC 11229.

### 4.2. Methodology of Experimental Research

**Figure 9:**
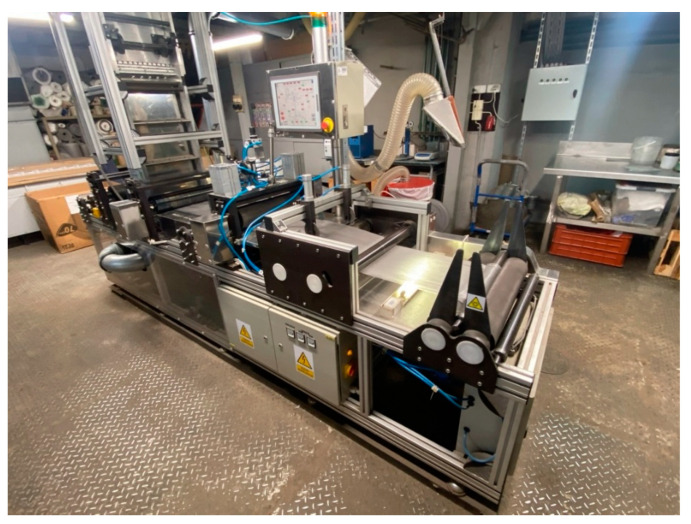
Laboratory station for spunbonded nonwoven fabric forming.

The process of forming nonwoven fabric by the spunbonded method was carried out on a laboratory bench designed and built by the Central Research and Development Center for Textile Machinery “Polmatex-Cenaro” (Poland) (Figure 9). The polymer in the form of granules was introduced into an extruder, where it was melted. In this form, it was fed through a filter to a pump, which pumped it to the filaments in the spinning head, forming strands of fibers. These passed through a wind tunnel, where they stretched and tightened. They then spread out in an irregular pattern on a moving conveyor mesh, forming a fleece. The fleece was fed further, leaving the conveyor, fused together by a crushing roller. Finally, with the help of a calender with heated rollers, the fibers were fused together under the influence of temperature, and the finished fleece was wound onto the pickup.

**Figure 10:**
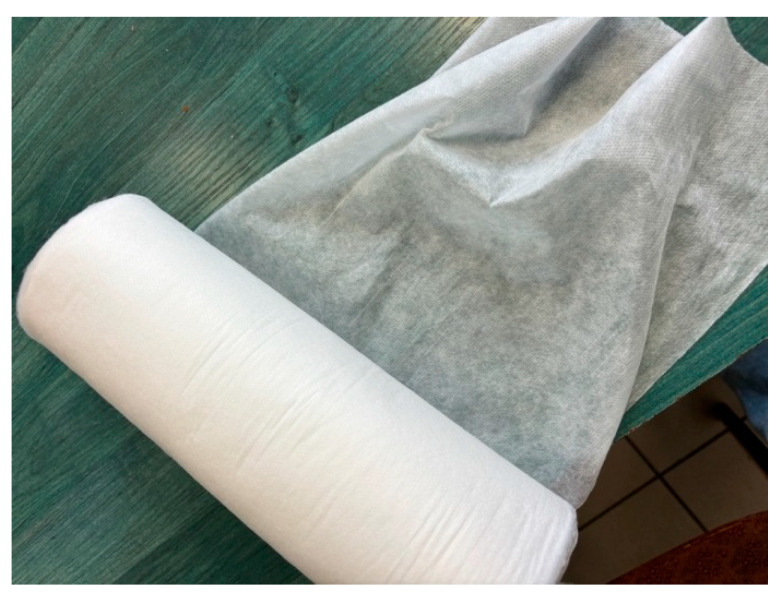
Spunbonded nonwoven fabric with PBS.

Figure 10 shows the resulting spunbond nonwoven fabric made from PBS. It is a durable nonwoven, resistant to stretching and tearing.

The obtained nonwoven fabric was subjected to metrological tests to determine its physical and mechanical properties. The test conducted included the following parameters: 1/thickness (mm), according to PN-EN ISO 9073-2:2002 [26]; 2/surface mass (g/m^2^), according to PN-EN 29073-1:1994 [27]; 3/breaking force (N), elongation at break (%), breaking stress (MPa), according to PN-EN 29073-3:1994 [28]. Determination of force, elongation and stress values was performed on an Instron model 5544 testing machine (USA).

**Figure 11:**
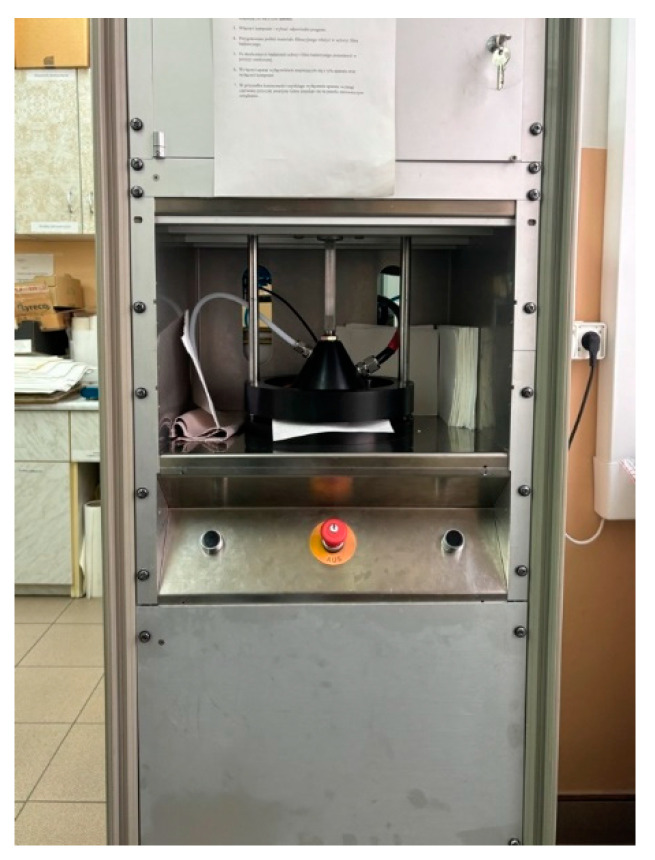
GRIMM test apparatus Type 7700 for testing the effectiveness of filter materials.

Applying the selected solution by surfacing to the nonwoven fabric consisted of soaking a 20.5 cm × 15 cm sample for five minutes and then allowing it to dry completely in air (temperature 24 °C, humidity 40%). Before and after surfacing, the samples were weighed to determine the degree of application of the apprettee. Testing of the efficiency of the nonwoven filter fabric was carried out on a GRIMM Type 7700 (Hamburg, Germany) test apparatus (Figure 11), based on the standard describing the total efficiency of the filter PN-EN 1822-1 [29]. The test consisted of placing a pre-cut sample, measuring 15 × 15 cm, in the test apparatus. The following samples were selected for the test: two single variants—left and right side of the nonwoven fabric, and two double variants—left over left and right over right. Based on the results, the most effective single and double variants were determined. These variants were then tested for composites with spunbond nonwoven, without and with aperture, and carbon nonwoven. Three measurements were taken for each sample. The nominal airflow rate was set at 5 cm/s. DEHS (diethyl sebacate) aerosol was used during the test. The evaluation of total filter efficiency was calculated from the concentration of particles upstream and downstream of the tested filter, using a probe. During the test, particles from a size greater than or equal to 0.3 µm to a size of 2 µm were counted. The test time for each sample was about 5 min. 

Verification of the biocidal treatment was carried out by testing the antibacterial activity of textiles, a quantitative test described in PN-EN ISO 20743:2021 [30]. The test consisted of inoculating nonwoven samples with the biocidal coating and control samples with a suspension of bacteria at a certain density and determining the number before and after incubation. The decrease in the number of cells after incubation to the control samples and samples before incubation indicates the biological activity of the samples. Before inoculation with bacteria, samples were sterilized by exposing each side to a UV lamp (10-min exposure). A suspension with a density of 1–3 × 10^5^ cfu/mL was used at a rate of 0.2 mL per sample. The prepared bacterial suspension with a density of 10^5^ cfu/mL was inoculated into the prepared samples. Half of the samples were rinsed by shaking in NaCl solution with Tween immediately after inoculation, and the remaining samples were incubated for 24 h at 37 °C, and then rinsed at the end of the incubation. From the resulting bacterial suspensions, appropriate dilutions were prepared in NaCl saline with peptone from each dilution, and depth cultures were performed on agar plates (TSA nutrient agar). After incubation, colonies on each plate were counted and the number of bacteria was calculated according to the formula:

(1) $$M=\frac{\sum C}{v\times n_{1}\times d}\cdot 20$$

where:

*M*—number of bacteria per sample;

*C*—sum of colonies on all plates from the counted dilution;

*v*—volume of culture applied to each plate in ml;

*n*_1_—number of plates from the counted dilution;

*d*—dilution index corresponding to the dilution counted;

20—number of milliliters of NaCl solution with Tween used to wash the bacteria out of the sample.

The value of antibacterial activity was calculated from the formula:

A = (lg C_t_ − lg C_0_) − (lg T_t_ − lg T_0_) (2)

where:

A—antibacterial activity value;

lg C_t_—the decimal logarithm of the number of bacteria on the control sample after 24 h incubation;

lg C_0_—the decimal logarithm of the number of bacteria on the control sample immediately after inoculation;

lg T_t_—the decimal logarithm of the number of bacteria on the sample with active agent after 24 h incubation;

lg T_0_—decimal logarithm of the number of bacteria on the sample with active agent immediately after inoculation.

## 5. Experimental Results

First, the results of metrological tests of the obtained spunbond nonwoven fabric were analyzed (Table 3). The material is relatively thin and light, demonstrating suitable properties for applications requiring lightness and adequate mechanical strength. In the longitudinal direction, the nonwoven fabric shows higher tensile strength than in the transverse direction. The elongation at break in the longitudinal direction is relatively low, meaning the nonwoven does not stretch significantly before breaking. In the transverse direction, however, the result is much higher, indicating greater flexibility of the nonwoven fabric in this direction. The tensile stress in the longitudinal direction is higher than in the transverse direction. The nonwoven fabric exhibits anisotropic mechanical properties.

The following variants of samples with and without the active layer were selected for testing:

- Sample 0—PBS nonwoven fabric;
- Sample 1.1—PBS nonwoven fabric with 100% thyme hydrosol;
- Sample 1.2—PBS nonwoven fabric with 50% thyme hydrosol + 50% distilled water;
- Sample 2.1—PBS nonwoven fabric with 100% witch hazel hydrosol;
- Sample 2.2—PBS nonwoven fabric with 50% witch hazel hydrosol + 50% distilled water.

**Figure 12:**
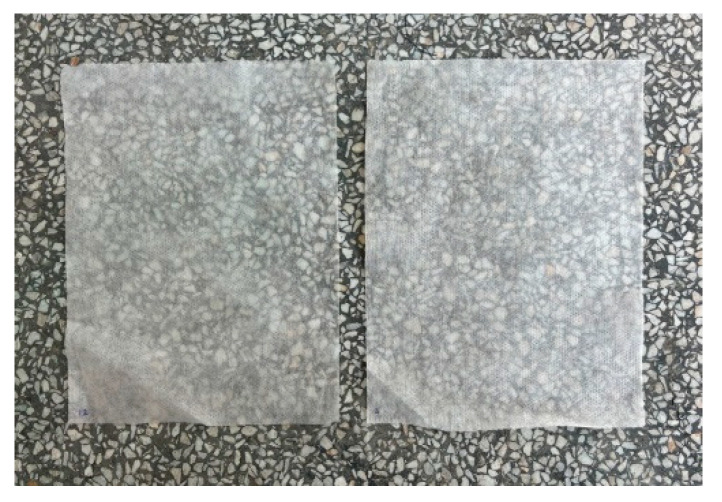
Sample before treatment (**left side**) and sample after treatment (**right side**).

Table 4 presents the degree of finish application on the selected samples. The samples treated in thyme solutions show a higher degree of finish application than the samples treated with witch hazel hydrosol solutions. Samples soaked in 100% solutions absorbed the substances better. The differences between the minimum and maximum values of finish application on the nonwoven fabric vary significantly. The results suggest that the degree of finish application could be more uniform. Figure 12 shows a sample of the nonwoven fabric before treatment (left side) and an example of a sample after treatment and drying (right side). The samples did not change in feel after the finish was applied, and their structure remained unchanged. Therefore, it was decided not to repeat the metrological tests for the modified samples.

Table 5 provides the legend for the symbols used for all the tested samples. In the next stage, the results were analyzed in terms of filtration efficiency (Table 6).

**Figure 13:**
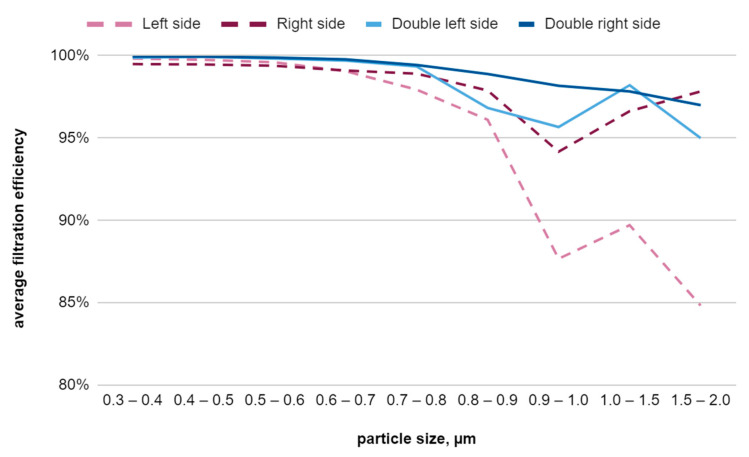
Graph of the average filtration efficiency of meltblown nonwoven fabric as a function of individual particle sizes.

Analysis of the filtration efficiency results revealed that the double sample, consisting of the right side of the nonwoven fabric for the meltblown nonwoven fabric in two variants, is the most effective. The single sample tested from the left side has the worst filtration efficiency. All samples show the highest results for smaller particles, as shown in Figure 13. Nonwovens arranged from the right side demonstrate better filtration efficiency for more penetrating, larger particles than the nonwoven samples arranged from the left sides. The single right-side sample and the double right-side sample were selected for further research.

**Figure 14:**
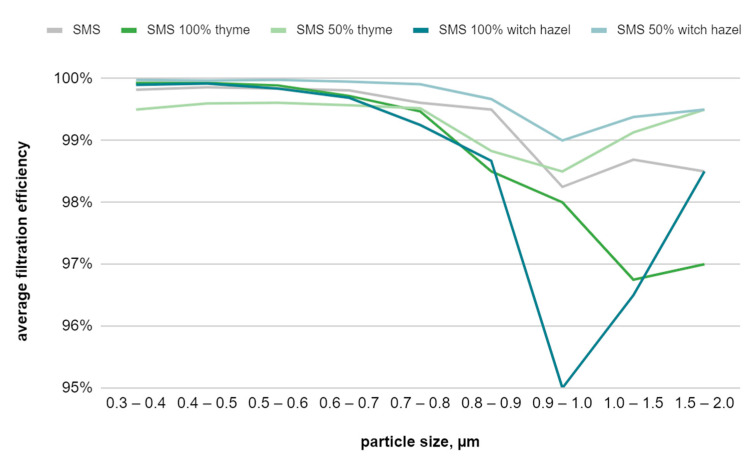
Filtration efficiency graph for SMS-type filtration composites as a function of particle size.

Table 7 presents the efficiency results for composites consisting of one layer of the right side of the meltblown nonwoven fabric and two layers of spunbond nonwoven fabric, with and without applied finish. As shown in Figure 14, the samples demonstrate better efficiency for smaller particles. The sample with a 50% witch hazel hydrosol solution finish exhibits higher filtration efficiency, while the sample with a 100% witch hazel hydrosol solution shows a lower value. Overall, samples treated with 100% solutions display lower values than those treated with 50% solutions, which also show the best results for larger particles. It was observed that the filtration efficiency of all samples increases compared to a single layer of meltblown nonwoven fabric, and the use of spunbond nonwoven fabric helps in retaining particles.

**Figure 15:**
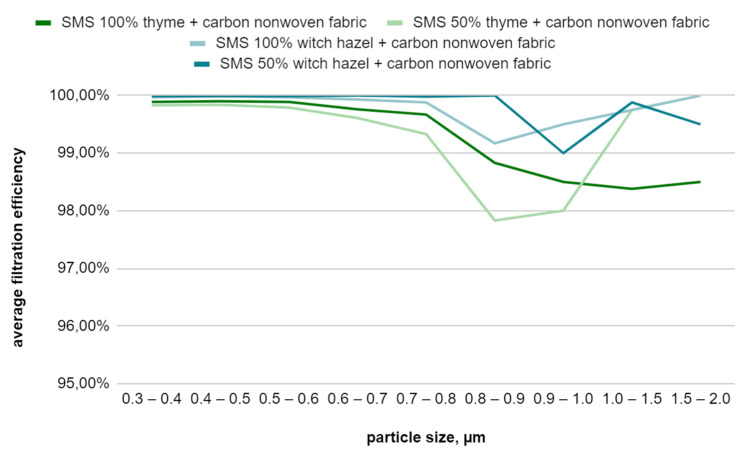
Filtration efficiency graph for the SMS-type filtration composite with an additional carbon fiber layer as a function of particle size.

The filtration efficiency results of the composite consisting of a single layer of the right-side meltblown nonwoven fabric, two layers of spunbond nonwoven fabric with finish, and carbon nonwoven fabric are presented in Table 8. The sample with a finish composed of 100% thyme hydrosol has the lowest values. The sample with spunbond nonwoven fabric treated with a 50% witch hazel hydrosol solution shows the best efficiency. All samples, except the 100% thyme sample, which has the weakest filtration for large particles, demonstrate good efficiency across the full spectrum of particle sizes (Figure 15). The filtration efficiency increased for all samples compared to the results of the composite without the carbon nonwoven fabric. This suggests that the additional composite element positively influences particle retention.

**Figure 16:**
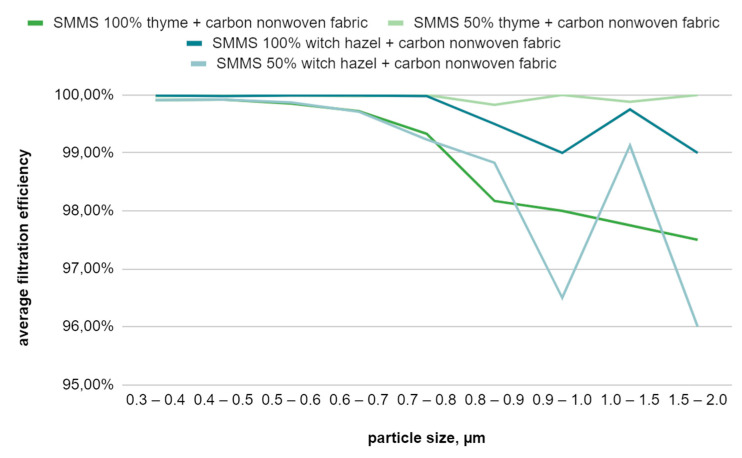
Filtration efficiency graph for the SMMS-type filtration composite with an additional carbon fiber layer as a function of particle size.

Table 9 presents the filtration efficiency results for a composite consisting of two layers of the right side of meltblown nonwoven fabric, two layers of spunbond nonwoven fabric with an antibacterial finish, and one layer of carbon fiber nonwoven fabric. The composite with spunbond nonwoven fabric treated with a 50% thyme hydrolate solution exhibits the highest efficiency, while the composite with spunbond nonwoven fabric treated with a 50% witch hazel hydrolate solution shows the lowest filtration efficiency. The efficiency values of all samples decreased compared to similar composites with only a single layer of meltblown nonwoven fabric, except for the sample with the 50% thyme hydrolate solution, for which this value increased. All samples demonstrate good filtration efficiency for small particles (Figure 16).

In the next stage, the air resistance values of the tested samples were analyzed. Table 10 presents the air resistance results for the individual samples. The lowest values were observed in the filtration composites consisting of spunbond nonwoven fabric with various finishes, one layer of meltblown nonwoven fabric, and carbon fiber nonwoven fabric. The highest values were recorded for double layers of meltblown nonwoven fabric, which in both cases reached 64 Pa. In every case studied, the addition of an extra layer of carbon fiber nonwoven fabric reduced the overall air resistance of the tested sample. Adding spunbond nonwoven fabric on the top and bottom of the sample increased the air resistance for the tested composite. It is noteworthy that each tested sample exhibited relatively low air resistance values for a filtration composite intended for an air purifier, indicating that air can flow more efficiently through the composite.

The additional layer of spunbond nonwoven fabric does not decrease the filtration efficiency of the meltblown nonwoven fabric; in fact, it can even increase its value. Moreover, the addition of carbon fiber nonwoven fabric to the composites positively affects both the filtration efficiency and the reduction of air resistance, allowing for easier airflow. Among all the tested samples, the composite consisting of a double right layer of meltblown nonwoven fabric, two layers of spunbond nonwoven fabric with a 50% thyme hydrolate solution finish, and carbon fiber nonwoven fabric (SMMS 50% thyme + carbon fiber nonwoven) exhibits the highest filtration efficiency values, both for small and large particles. Therefore, it can be concluded that this composite demonstrates filtration properties at the level of an H12 class filter, which is sufficient quality for use in domestic air purifiers [29].

In the next stage, the antimicrobial performance results of the nonwoven fabric samples were analyzed, which were evaluated according to the criteria provided in Table 11. 

The results of the evaluation of antimicrobial activity for *Escherichia coli* bacteria are shown in Table 12. Their number on each test sample increased after a 24 h incubation. For the samples surfaced with hydrolates, these values are lower than for the control sample. None of the tested samples with an applied dressing are within the limits of the standard [30]. The highest values of antibacterial activity are shown by the sample with a 50% solution of witch hazel hydrolate. The sample with 100% thyme hydrolate has the weakest antibacterial activity. 

Table 13 shows the results of evaluating the antibacterial activity of the nonwoven spunbond fabric with and without the applied apprettee. In each case tested, the number of *Staphylococcus aureus* bacteria increased after a 24 h incubation. For both samples with thyme hydrolate and 100% witch hazel hydrolate, the number was lower than the value for the control sample. On the sample with 50% witch hazel hydrolate, the number of bacteria after incubation was higher than the value for the control sample. The results obtained do not meet the values given in the standard. The highest result of antimicrobial activity evaluation has a nonwoven fabric with 50% thyme hydrolate.

All samples tested showed low biocidal properties. Samples 1.1, 1.2 and 2.1 showed a slight decrease in microorganisms. Further testing would be necessary to achieve the desired values of antibacterial activity. One option would be to improve the apertures used, by using essential oils to increase the concentration of the active ingredient. Another approach could be to use a modifier to increase the absorptive properties of the nonwoven fabric, which would allow for increased application of the textile finish.

## 6. Summary

The form of the diffuser was designed based on computer simulations, which allowed a thorough analysis of airflow. It was investigated how the air spreads depending on the geometry used, and its speed and direction were analyzed. It was concluded that the holes located at the top of the lamp provide better air distribution to the environment and do not block the airflow. Plans are underway to further explore diffuser geometry to optimize airflow in different environments by testing more variants and configurations in real-world scenarios. Experimental and numerical studies will evaluate the impact of different scrubber sizes on scrubber deployment and performance in indoor environments. The article also presents the possibility of an innovative filter composite with biocidal properties. To create it, a layer made of nonwoven spunbond fabric was surfaced with hydrolates to give it bactericidal properties. The composite of spunbond nonwoven fabric, meltblown nonwoven fabric and nonwoven carbon fabric was then tested for filtration efficiency. The study showed that the entire SMMS 50% thyme + nonwoven carbon composite showed the highest values of filtration efficiency, both for small and large particles. Unfortunately, the composite did not meet the antibacterial activity values given in the standards. Further work is planned leading to a solution to this problem. The use of hydrolat or essential oils with higher concentrations of active ingredients to improve the antibacterial performance of the filter will be considered.

## Figures and Tables

**Table 1:** Basic parameters of nonwoven meltblown fabric.

Parameter	
Thickness of nonwoven fabric, mm	0.24
Surface weight, g/m^2^	20–25
Tensile strength in longitudinal direction, N/5 cm	11
Elongation at break in longitudinal direction, %	30
Tensile strength in the transverse direction, N/5 cm	11
Elongation at break in the transverse direction, %	40

**Table 2:** Basic parameters of nonwoven carbon fabric.

Parameters	
Thickness of nonwoven fabric, mm	4
Surface weight, g/m^2^	150

**Table 3:** Results of metrological tests of spunbond nonwoven fabric made from PBS.

Tested Parameter	Result	Measurement Uncertainty
Nonwoven fabric thickness, mm	0.26	±0.01
Surface weight, g/m^2^	29.80	±0.30
Breaking force in the longitudinal direction, N	5.91	±0.53
Elongation at break in the longitudinal direction, %	7.96	±0.62
Breaking stress in the longitudinal direction, MPa	0.52	±0.04
Breaking force in the transverse direction, N	3.99	±0.41
Elongation at break in the transverse direction, %	24.80	±8.89
Breaking stress in the transverse direction, MPa	0.35	±0.03

**Table 4:** Results of the degree of finish application.

Sample No	Minimum Value, %	Maximum Value, %	Average, %
Sample 1.1	0.55	0.97	0.65
Sample 1.2	0.47	0.93	0.60
Sample 2.1	0.45	0.71	0.52
Sample 2.2	0.16	0.43	0.32

**Table 5:** Legend of markings of tested samples.

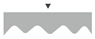	Left side of meltblown nonwoven fabric	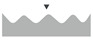	Right side of meltblown nonwoven fabric
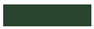	Sample 1.1—spunbond nonwoven fabric with 100% thyme hydrolate	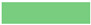	Sample 1.2—spunbond nonwoven fabric with 50% thyme hydrolate
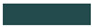	Sample 2.1—spunbond nonwoven fabric from 100% witch hazel hydrolate	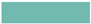	Sample 2.2—spunbond nonwoven fabric with 50% witch hazel hydrolate
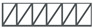	Carbon nonwoven fabric	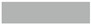	Spunbond nonwoven fabric

**Table 6:** Performance results of meltblown nonwoven filter fabric.

Composite Composition	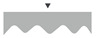	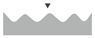	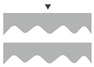	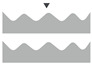
	Single Left	Single Right	Double Left-Left	Double Right-Right
Average effectiveness	94.94%	98.10%	98.27%	98.99%

**Table 7:** Efficiency results of SMS-type filtration composites.

Composite Composition	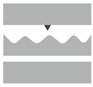	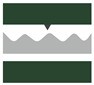	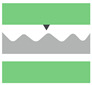	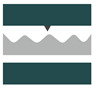	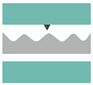
	SMS	SMS 100% Thyme	SMS 50% Thyme	SMS 100% Hazel	SMS 50% Hazel
Average efficiency	99.32%	98.80%	99.31%	98.59%	99.70%

**Table 8:** Efficiency results of the SMS-type filtration composite with an additional layer of carbon nonwoven fabric.

Composite Composition	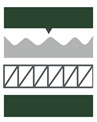	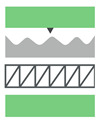	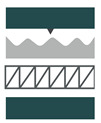	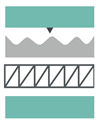
	SMS 100% Thyme + Carbon Nonwoven Fabric	SMS 50% Thyme + Carbon Nonwoven Fabric	SMS 100% Hazel + Carbon Nonwoven Fabric	SMS 50% Hazel + Carbon Nonwoven Fabric
Average efficiency	99.26%	99.33%	99.79%	99.82%

**Table 9:** Filtration efficiency results of the SMMS-type filtration composite with an additional carbon fiber layer.

Composite Composition	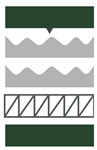	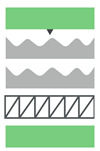	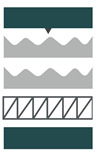	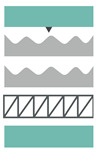
	SMMS 100% Thyme + Carbon Nonwoven Fabric	SMMS 50% Thyme + Carbon Nonwoven Fabric	SMMS 100% Hazel + Carbon Nonwoven Fabric	SMMS 50% Hazel + Carbon Nonwoven Fabric
Average efficiency	98.91%	99.96%	99.69%	98.79%

**Table 10:** Air resistance results for the filtration of nonwoven fabric and filtration composites.

Tested Sample	Air Resistance, Pa
Single left	35
Single right	35
Double left	64
Double right	64
SMS	40
SMS 100% Thyme	43
SMS 50% Thyme	42
SMS 100% Hazel	42
SMS 50% Hazel	42
SMS 100% thyme + carbon nonwoven fabric	34
SMS 50% thyme + carbon nonwoven fabric	33
SMS 100% hazel + carbon nonwoven fabric	33
SMS 50% hazel + carbon nonwoven fabric	32
SMMS 100% thyme + carbon nonwoven fabric	53
SMMS 50% thyme + carbon nonwoven fabric	54
SMMS 100% hazel + carbon nonwoven fabric	54
SMMS 50 hazel + carbon nonwoven fabric	53

**Table 11:** Criteria for evaluating antibacterial activity according to the PN-EN ISO 20743:2021 standard [30].

Antibacterial Effectiveness	Antibacterial Activity Value
low	A < 2
significant	2 ≤ A < 3
strong	A ≥ 3

**Table 12:** Antibacterial activity for *Escherichia coli* ATCC 11 229.

Samples	Incubation Time, h	Number of Bacteria, jtk/pr	Antimicrobial Activity Value, A		Value of Growth
Control sample	0	8.5 × 10^3^			4.3
	24	1.6 × 10^8^			
Sample 1.1 100% thyme hydrolate	0	1.5 × 10^4^	0.13		4.2
	24	2.1 × 10^8^			
Sample 1.2 50% thyme hydrolate	0	1.5 × 10^4^	0.20		4.1
	24	1.8 × 10^8^			
Sample 2.1 100% hazel hydrolate	0	2.2 × 10^4^	0.36		3.9
	24	1.8 × 10^8^			
Sample 2.2 50% hazel hydrolate	0	2.4 × 10^4^	0.40		3.9
	24	1.8 × 10^8^			

**Table 13:** Antibacterial activity for *Staphylococcus aureus* ATCC 6538.

Samples	Incubation Time, h	Number of Bacteria, jtk/pr	Antimicrobial Activity Value, A		Value of Growth
Control sample	0	1.3 × 10^3^			3.7
	24	7.1 × 10^6^			
Sample 1.1 100% thyme hydrolate	0	1.4 × 10^4^	0.26		3.5
	24	4.2 × 10^7^			
Sample 1.2 50% thyme hydrolate	0	9.7 × 10^3^	0.45		3.3
	24	1.9 × 10^7^			
Sample 2.1 100% hazel hydrolate	0	4.9 × 10^3^	0.31		3.4
	24	1.3 × 10^7^			
Sample 2.2 50% hazel hydrolate	0	1.4 × 10^3^	−0.20		3.9
	24	1.2 × 10^7^			

## Data Availability

The data presented in this study are available in the databases of the authors at the Lodz University of Technology.
